# Nanofibril scaffold assisted MEMS artificial hydrogel neuromasts for enhanced sensitivity flow sensing

**DOI:** 10.1038/srep19336

**Published:** 2016-01-14

**Authors:** Ajay Giri Prakash Kottapalli, Meghali Bora, Mohsen Asadnia, Jianmin Miao, Subbu S. Venkatraman, Michael Triantafyllou

**Affiliations:** 1 School of Mechanical & Aerospace Engineering, Nanyang Technological University, 50 Nanyang Avenue, Singapore 639798; 2 Center for Environmental Sensing and Modeling (CENSAM) IRG Singapore-MIT Alliance for Research and Technology (SMART) Centre, 3 Science Drive 2, Singapore 117543; 3 School of Material Science and Engineering, Nanyang Technological University, 50 Nanyang Avenue, Singapore 639798; 4 School of Electrical, Electronic and Computer Engineering, University of Western Australia, Perth, Western Australia 6009, Australia; 5 Department of Mechanical Engineering, Massachusetts Institute of Technology, 77 Massachusetts Avenue, Cambridge, MA 02139.

## Abstract

We present the development and testing of superficial neuromast-inspired flow sensors that also attain high sensitivity and resolution through a biomimetic hyaulronic acid-based hydrogel cupula dressing. The inspiration comes from the spatially distributed neuromasts of the blind cavefish that live in completely dark undersea caves; the sensors enable the fish to form three-dimensional flow and object maps, enabling them to maneuver efficiently in cluttered environments. A canopy shaped electrospun nanofibril scaffold, inspired by the cupular fibrils, assists the drop-casting process allowing the formation of a prolate spheroid-shaped artificial cupula. Rheological and nanoindentation characterizations showed that the Young’s modulus of the artificial cupula closely matches the biological cupula (10–100 Pa). A comparative experimental study conducted to evaluate the sensitivities of the naked hair cell sensor and the cupula-dressed sensor in sensing steady-state flows demonstrated a sensitivity enhancement by 3.5–5 times due to the presence of hydrogel cupula. The novel strategies of sensor development presented in this report are applicable to the design and fabrication of other biomimetic sensors as well. The developed sensors can be used in the navigation and maneuvering of underwater robots, but can also find applications in biomedical and microfluidic devices.

We often resort to nature for inspiration in order to develop designs that emulate the function and performance of animals. Engineers have invented flapping flyers that can develop large lift forces like the insects do^1^, created artificial leafs that can generate chemical energy from sunlight through photosynthesis^2^, developed highly maneuverable drones by mimicking the basic principles of bird flight^3^, fabricated devices that can extract energy like a fish performing Karman gaiting^4^, harnessed genetically encoded logic to reprogramme living systems^5^, designed surfaces that can harvest water like a desert beetle^6^, fabricated prosthetic limbs with artificial skins that sense like real skin, etc^7^. Evolution enables biological organisms to develop optimal solutions for specific functions, which can be studied and emulated. Certain animal sensors portray complex nanostructures, and utilize a variety of sensing principles to exhibit enhanced sensitivity, and impressive sensing performance, which in certain cases exceeds the performance of man-made systems. For instance, to permit foraging in dark environment, the blind cavefish developed taller superficial neuromasts^8^ with encapsulated cupular fibrils, which resulted in enhanced sensitivity to flow sensing^9^. The canal channel of the canal neuromasts of certain fish species developed constrictions which resulted in attentuation of low frequency flows and amplification of high frequency flows^10^. The active somatic mutility of the inner ear hair bundles not only amplifies acoustic signals by hundred times, but also fine-tunes frequency selectivity and broadens the dynamic range^11^. In nature, we can find fine designs of miniature biological sensors that work efficiently and accurately, and are long lasting. Efficiency, accuracy, sensitivity, and reliability in artificial sensors and sensing systems are exactly what most modern researchers are grappling with. Therefore, translating nature-based engineering solutions to artificial manmade technologies could lead to innovative solutions to solve crucial problems.

Blind cavefish, that live and function in deep-waters, is bestowed with a set of flow sensors called neuromasts, which enable them to detect minute water flow variations down to the order of 18–38 μm/s^12^ and 1 cm/s^13^ for oscillatory flows of frequency range 10–20 Hz and steady-state flows respectively. Using the neuromasts, that are spatially distributed on the body, fishes can accomplish tasks like ‘hydrodynamic vision’^14^, energy-efficiency in maneuvering^15^,^16^, and schooling^14^, and can navigate with great agility under hydrodynamically challenging conditions.

The superficial and canal neuromasts function in a ‘division of labour’ principle (Fig. 1a–d) and perform sensing of various hydrodynamic flow phenomena experienced by the fish. The superficial neuromasts are directly exposed to water and best respond to direct current and low frequency flows encountered by the fish (<30 Hz)^10^. The canal neuromasts that are present in sub-dermal canals are exposed to the flow through a series of pores, and can sense pressure differences between pores; hence, they can detect disturbances and oscillatory flows of higher frequencies (30–150 Hz)^17^. Apart from the variations in physical dimensions, shape and functionality, the morphology and basic sensing principles are similar in both sensors^18^. The neuromasts of most fishes consist of three major components – the cupula, cupular fibrils and the hair cells (Fig. 1a). The cupulae are made up of soft gelatinous glycoprotein material^19^ and are 100–1000 μm in height and 100 μm in diameter^20^(the dimensions vary depending upon species and age). The cupula not only forms a mechanical connection between external flow and mechanosensing hair cells but also enhances the signal transmission to the hair cells in several ways. The large surface area of cupula enhances the drag force exerted by flow and thereby increases the bending of the embedded cilia^14^. It is also hypothesized that the enhanced friction factor that is associated with the hydrophilicity and permeability of cupular material enhances the signal absorption^19^. The inertial mass of cupula leads to a low-pass filtering nature of the superficial neuromast leading to its less responsiveness to flows beyond a cut-off frequency of 10–60 Hz^21^. The cupular fibrils embedded within the cupula of superficial neuromasts of blind cavefish are believed to support the structural network of the soft cupula allowing it to grow to heights that extend beyond the boundary layer generated by the flow^15^,^16^. The cupular fibrils are also believed to aid in coupling the hair cells to the cupula thus allowing an enhanced transmission of energy from the cupula to the sensing hair cell sensors^20^,^21^,^22^.

The artificial microelectromechnical systems (MEMS) sensors we have developed embrace the structural design and sensing principles of the neuromast sensors and hence attain their sensing performance. We present biomimetic all-polymer MEMS flow sensors that demonstrate enhanced sensitivity due to the presence of cupulae inspired by the superficial neuromasts on the fish. We introduce the development of an electrospun nanofibril canopy-like structure that assists the formation of a prolate spheroid-shaped hydrogel cupula. The nanofibril canopy acts as a scaffold during the drop-casting and gets encapsulated within the cupula after its formation and wetting. From a practical view point, availability of such miniaturized yet robust, low-powered yet ultrasensitive, and light-weight yet low-cost, and surface-mountable flow sensors would offer significant benefits in flow sensing on undersea vehicles. Knowledge of information of the flows on the surface of a vehicle can assist in treading towards- energy-efficient maneuvers, passive near-field object detection and improved control of the vehicle– skills that undersea animals naturally achieve. In the past, researchers developed bio-inspired pressure sensors and biomimetic flow sensors inspired by the lateral-line of blind fish^19^,^23^,^24^,^25^,^26^,^27^,^28^,^29^,^30^,^31^,^32^,^33^,^34^. Chen N. *et al.* developed artificial hair cells consisting of a silicon cantilever beam with a high aspect-ratio SU-8 cilium attached to it at the distal end^19^. They demonstrated through steady-state flow sensing experiments that these silicon hair cell sensors were able to achieve ultrahigh sensitivity to flow velocity sensing. For steady state flow sensing experiments conducted in similar experimental conditions, the polymer naked hair cell sensors (with out cupula dressing) we developed in this work demonstrated a sensitivity higher than those reported in literature^19^. There have been attempts to develop MEMS hair cell sensors which utilize a variety of sensing principles in order to attain the functionality of the biological hair cells. Yang Y. C. *et al.* developed 3D microstructures with nickel filaments that utilize thermal hot wire anemometry sensing to image hydrodynamic flow^33^. Klein A. *et al.* developed hair cells with optical fibers which sense velocity depending on the modulation of light guided through the fiber^23^. Kottapalli and Asadnia *et al.* developed self-powered MEMS hair cell sensors that featured piezoelectric membranes at the base of the long hair cells which interact with flow^26^,^27^. McConney *et al.* demonstrated that biologically inspired artificial cupulae enhanced the sensitivity of the superficial neuromast inspired flow sensors as compared to the ‘naked’ hair cell sensors that they developed by 2.5 times^5^. Most of the experiments were conducted employing a vibrating sphere (dipole) stimulus to evaluate the object localization^23^,^24^,^25^,^26^,^27^, flow sensing^28^,^29^,^30^,^31^,^32^ and hydrodynamic wake detection^33^,^34^ performance of the sensor arrays. As compared to the biomimetic cupulae developed in the past, the nanofibril scaffold introduced in this report establishes a well-controlled cupula fabrication method, enhances sensitivity by forming a prolate spheroid shaped cupula, enhances device to device fabrication repeatability and improves the integrity of the cupula to the embedded polymer hair cell, thereby improving the flow sensing performance of the sensor. Moreover, most flows sensed by superficial neuromasts are steady-state flows^10^,^12^,^13^, and employing a dipole stimulus to analyse the sensitivity enhancement of the superficial neuromast-inspired flow sensors may not be appropriate^35^. We report the development of a much softer hyaulronic acid methacrylic anhydride (HA-MA) hydrogel cupula, optimized to have a closest match of Young’s modulus to the biological cupula^36^. Comparative experimental studies, that analyse the sensitivity of the cupula encapsulated sensors, to naked hair cell sensors, is demonstrated by testing their response to a wide-range of steady-state flows. The threshold detection limits achieved by our artificial cupula sensor (18 mm/s) also compare favourably to those of the biological neuromast in fish investigated in the past (10 mm/s for steady-state flows)^12^,^13^.

## Results

### Development of MEMS artificial cupula sensor

**Figure 2:**
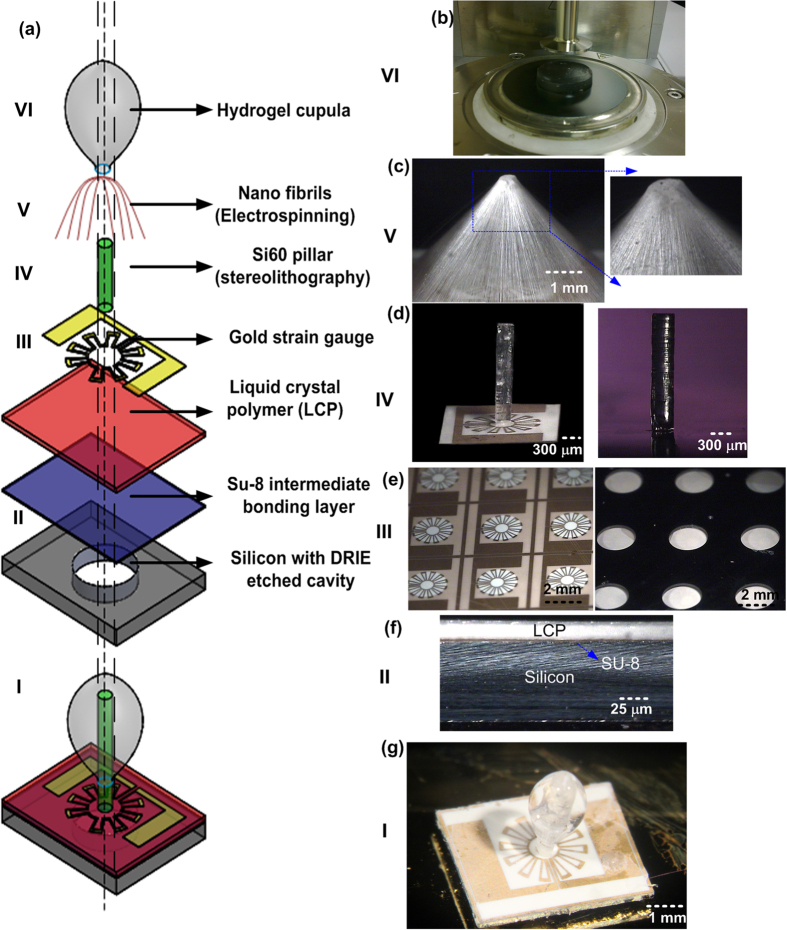
Fabrication process flow to form an artificial MEMS superficial neuromast: (**a**) A schematic showing an exploded view of various fabrication processes and the materials used in fabrication. (**b**) A photograph showing 25 mm disc of swollen HA-MA hydrogel undergoing rheology test. (**c**) Electrospun nanofibril scaffold showing aligned nanofibers bridging the distal tip of the hair cell to the perimeter of the sensor. These nanofibrils act as a scaffold during the hydrogel formation and also enhance the mechanical strength of the hydrogel. (**d**) Naked hair cell sensor (without the cupula dressing) featuring a high-aspect ratio stereolithographically printed polymer hair cell (**e**) Top and bottom views of LCP sensing base. 25 μm thick LCP membrane is defined by etching the silicon support wafer to which it is bonded (**f**) An optical image of the bond interface between silicon and LCP using an intermediate SU-8 layer (**g**) The artificial cupula sensor after forming the hydrogel cupula. (Image (**b**) was taken by Meghali Bora. Images (**c–g**) were taken by Ajay Giri Prakash Kottapalli and Mohsen Asadnia. Images were edited by Ajay Giri Prakash Kottapalli and Mohsen Asadnia).

The fabrication of the MEMS artificial cupula flow sensor mainly constitutes of 6 major steps – Liquid crystal polymer (LCP)-silicon bonding with intermediate SU-8 adhesion layer, patterning serpentine strain gauges on LCP top side with aligned DRIE cavities on the silicon bottom side, stereolithography to form the hair cells, hair cell positioning on LCP membrane, electrospun nanofibril scaffold formation and drop-casting process to form cupula-like structure. Figure 2a shows schematically an exploded view of the entire fabrication process to form the MEMS sensor. Figure 2b–g show the optical images of the sensor at various stages of fabrication. Our biomimetic flow sensor features a hydrogel cupula, 2700 μm tall and 1000 μm in diameter that extends beyond the flow-generated boundary layers (detailed design presented in supplementary information S1). The hydrogel cupula couples the drag force induced by fluid-structure interaction to the hair cell. The nanofibril matrix encapsulated within the cupula adheres the cupula to the hair cell, especially in the presence of strong flows. High-aspect ratio polycarbonate hair cells, 2700 μm tall and 350 μm in diameter, are fabricated by high-resolution stereolithography. The hair cells stand vertically with one end at the base connected to a 25 μm thick LCP membrane and are free to move at the distal end that protrudes into the flow. The LCP membrane is patterned with serpentine shaped strain gauges that surround the hair cell. An LCP membrane of 2 mm diameter is realized by bonding a 25 μm thick film of LCP to a silicon wafer and etching a through-hole in silicon by deep reactive ion etching (DRIE). This work significantly expands our previously reported study on design and fabrication of the polymer hair cell sensors^37^,^38^. Since the cupula and the cupular fibers synergistically enhance the flow sensing performance of the hair cells^20^, the major contribution of this work is to develop the nanofibril scaffold-assisted artificial cupula formation on the hair cell and then experimentally validate the sensitivity enhancement due to the presence of artificial cupula as compared to the naked hair cells.

### Material characterization of Hyaulronic acid (HA)-based synthetic cupula

**Figure 3:**
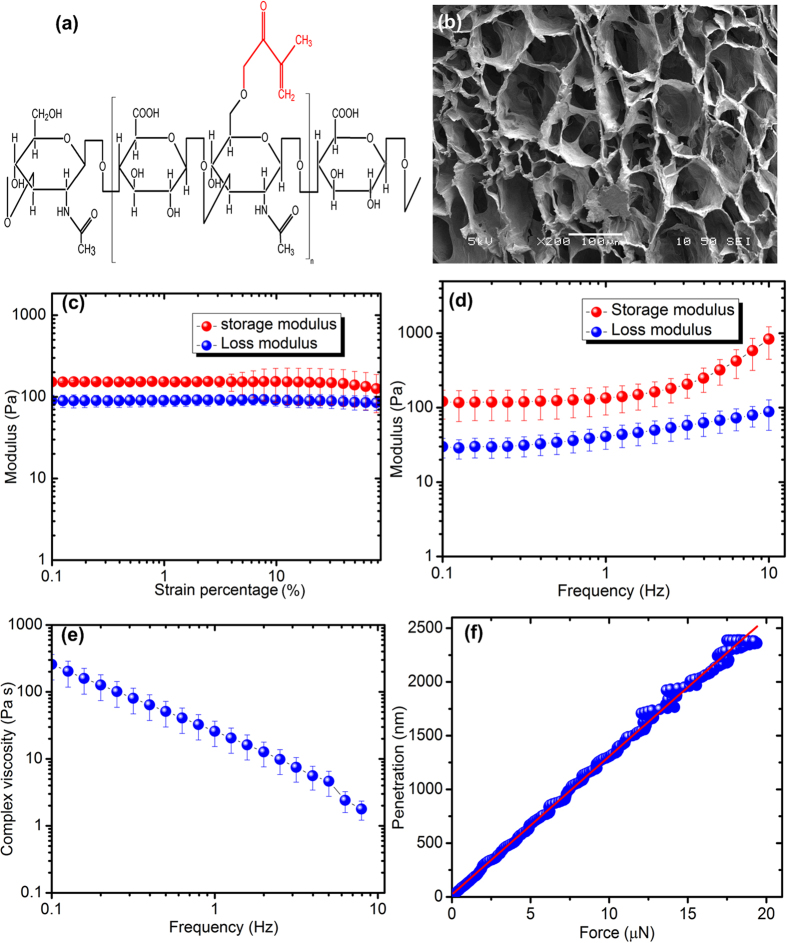
Characterization of the material properties of 2% HA-MA hydrogel with 0.1% initiator concentration that were moulded into 25 mm diameter samples (**a**) Chemical structure of HA-MA macromer (the attached chemical moiety is marked in red) (**b**) SEM image of cross-section of the hydrogel showing it’s porous structural organization (**c**) Rheology strain sweep of hydrogel showing the linear behaviour over strain percentage (**d**) Storage and loss modulus as a function of frequency (**e**) complex viscosity as a function of frequency (**f**) Nanoindentation analysis to determine the E of the hydrogel. (Image (**b**) was taken by Meghali Bora. Images were edited by Ajay Giri Prakash Kottapalli).

The Young’s modulus (E) of biological cupula (superficial cupulae of zebra fish) was reported by McHenry and van Netten to be 10–100 Pa^36^. In this work, a polymer cupula of comparable mechanical properties was synthesized using methacrylic acid modified hyaulronic acid hydrogel. Hydrogels are crosslinked networks of hydrophilic polymers capable of holding large volumes of water (Fig. 3a and detailed HA-MA structure is shown in supplementary information S2). The swelling or water holding capacity of hydrogels is a net resultant of two opposing forces that balance out after the gel reaches swelling equilibrium. The expansion forces namely; polymer-water interaction, electrostatic, and osmosis, serve to expand the polymer network while elastic forces stemming from polymer cross-linking points prevent its infinite solubility. The cross-linking reaction variables, that include macromer concetration, initiator concentration, and UV exposure time, were optimized to obtain the desired crosslinking density. From rheological and nanoindentation experiments, 0.1% Irgacure 2959 was chosen as the suitable initiator concentration for crosslinking that provided a close match of Young’s modulus to the biological cupula. Solutions of 2% HA-MA with 0.1% I2959 were exposed to UV light at 365 nm for 10 mins (Karl Suss MA6 mask aligner) for cross-linking followed by incubation of these gels in an aqueous medium for 24 h to reach swelling equilibrium. Higher initiator concentrations result in stiffer cupula due to higher cross-linking density until a limit when no reactive groups are available for further gelation and 100% cross-linking has been achieved. The swollen hydrogels were cut longitudinally and transversely, rapidly frozen at −80 **°**C, and lyophilized for 2 h for SEM sample preparation. The surface of HA-MA hydrogel was observed to be smooth from SEM images and pores could be observed on the surface as well as in the cross-section of samples (Fig. 3b).

In order to perform rheology and nanoindentation experiments, and swelling ratio and water percentage calculations, samples of 2% HA-MA hydrogel with 0.1% initiator concentration were moulded into 25 mm diameter discs. The samples were crosslinked and swollen as described above. Mechanical properties of HA-MA hydrogels were characterized using dynamic oscillatory shear tests in a stress-controlled rheometer (Physica MCR 501, Anton Parr, USA). All the tests were performed using parallel plate measuring system (PP25/TGSN 6539, diameter 25 mm) with zero normal force on the samples to minimize any damage. A strain sweep test, conducted at an angular frequency of 10 rad/s, showed a linear behaviour throughout the range of % strain rates (Fig. 3c). 100% strain rate was then selected to conduct the frequency sweep test to determine the storage modulus, loss modulus, and complex viscosity over the frequency range of 0.1 to 10 Hz. It can be observed that even at high strain rate, the values of elastic moduli do not vary significantly except at frequencies higher than 5 Hz (Fig. 3d) that can be considered as a usual trend of viscoelastic materials far from glass transition temperature^39^. The storage modulus of hydrogels varied from 15–45 Pa with lower modulus at higher frequencies. The complex viscosity of HA-MA hydrogel decreased linearly with increasing frequency (Fig. 3e). This behaviour could be attributed to the dynamics of mechanical energy dissipation in entangled networks^40^. Nanoindentation experiments conducted on five hydrogel samples indicated an average Young’s modulus of 7 Pa (Fig. 3f).

Percentage of water content in the HA-MA hydrogels, determined by weighing the sample before and after reaching the swelling equilibrium process, was found to be 82%. Mass swelling ratio (Q_M_) and was found to be ~36. The volumetric swelling ratio (Q_*v*_), mesh size, and crosslinking density (number of cross-links per unit volume) of HA-MA hydrogels, obtained using Flory-Rehner calculations^32^,^41^, were ~414 nm, 414 nm and 4.55 × 10^5 ^g/mol, respectively (details of calculations are provided in supplementary information S3).

### Electrospun nanofibril scaffold to form the cupula structure

**Figure 4:**
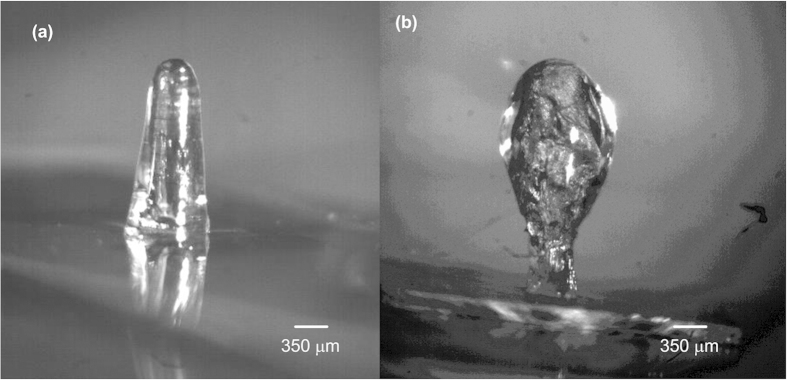
Optical microscopic images of an artificial hydrogel cupula: (a) Hydrogel drop-casted directly on the hair cell without the use of the nanofibril scaffold forms a semi-ellipsoid shaped cupula that creeps due to gravity onto the sensing membrane and forms the maximum diameter at the base. (**b**) Cupula formed using a nanofibril scaffold has a prolate spheroid shape. The nanofibril scaffold prevents the hydrogel from reaching the membrane and ensures that the cupula has a maximum diameter in a location that extends into the flow beyond the boundary layer. (Images (**a,b**) were taken and edited by Ajay Giri Prakash Kottapalli).

A canopy-like nanofibril pyramid was formed around the hair cell by electrospinning (Fig. 2c). The nanofibril structure acts as a scaffold during the drop-casting process assisting the HA-MA solution as it creeps along the hair cell due to gravity. More importantly, it also ensures the formation of a prolate spheroid-shaped cupula (Fig. 2g), wherein, the surface area exposed to the flow is maximized while the base of the hydrogel forms no contact with the LCP sensing membrane. These two factors majorly contributed to the enhancement in sensitivity that we obtained. The diameter of the hydrogel cupula reaches its maximum at a region above the mid-point along the length of the extended cupula. Therefore, the cupula exposes a maximum increase in surface area to the flow away from the boundary layer generated by the interface of the LCP membrane and the fluid flow. Absence of any contact with the LCP sensing membrane means that the hydrogel structure would not affect the torque-generated bending of the sensing membrane. This is in contrast with the direct drop-casting methods employed in the past that resulted in the formation of semi-ellipsoid shaped cupulae with the base of hydrogel structure supported on MEMS sensing structure^15^,^32^,^35^,^39^. Figure 4 shows the optical microscope images, which compare the structures of cupulae, formed after drop-casting process conducted directly on the hair cell to the ones formed using a nanofibril scaffold. As compared to direct drop-casting to form the cupula, the nanofibril scaffold assisted cupula formation illustrated in this report resulted in flow sensors with enhanced sensitivity.

**Figure 5:**
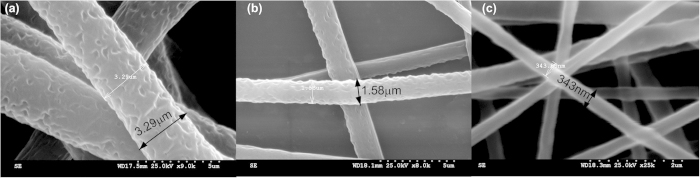
Scaffold optimization: Electrospun PCL nanofibers of three different diameters (a) 3 μm (b) 1.5 μm and (c) 350 nm formed by varying the spinning parameters. (Images (**a–c**) were taken and edited by Ajay Giri Prakash Kottapalli).

The nanofibril scaffold was developed through careful optimization of electrospinning process to form a canopy of aligned fibres connecting the distal tip of the hair cell to the perimeter of the LCP sensor base (Fig. 2c) (more images provided in the supplementary information S4). Nanofibers were formed using a solution of 11.7% (w/w) polycaprolactone (PCL) (MW 80,000 Da) in chloroform (CHCL_3_). LCP is electrically non-conducting and thus a 500 μm gold line was patterned along the perimeter of the sensor base to form the conducting region for selective attachment of the nanofibers. The protrusion of hair cell and the gold perimeter of the LCP sensor together assisted in the formation of a uniform and aligned network of fibers. The diameter of the nanofibers is crucial in determining the strength of the scaffold. Thus, the spinning parameters were varied to optimize the diameter of the fibers in the resulting scaffold that would be suitable for hydrogel drop-casting process. These parameters are such as precursor polymer feed rate, needle diameter, electric field, sample needle distance and spinning time, control the type, quality and density of fibrous network formed. In this report, we demonstrated the scaffold optimization using three different fiber diameters (Type i – 3 μm, Type ii - 1.5 μm and Type iii - 350 nm) as described in Table 1. SEM images of the three types of fibers varying in their diameter are shown in Fig. 5.

**Figure 6:**
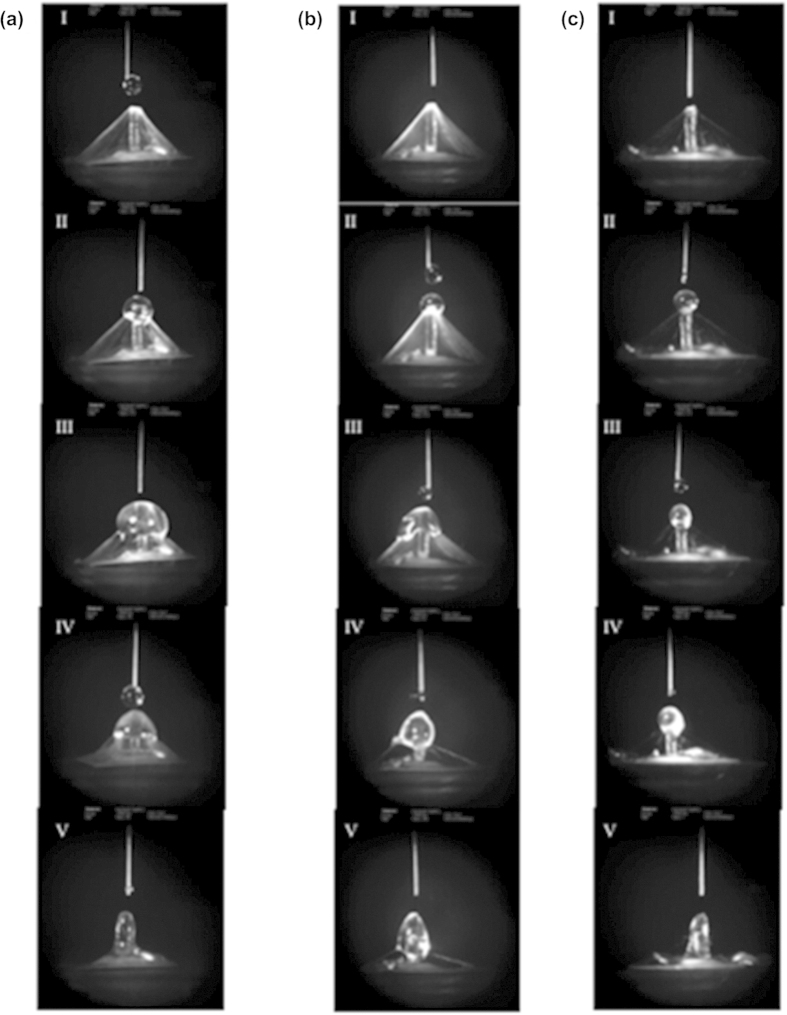
A series of high-speed camera images (order sequence numbered as I, II, III, IV and V) taken at different time instants during the hydrogel drop-casting process. (**a**) Drop-casting on type i nanofibril pyramid; (**b**) drop-casting on type ii nanofibril pyramid; (**c**) drop-casting on type iii nanofibril pyramid. (Images (a–c) were taken and edited by Ajay Giri Prakash Kottapalli).

**Figure 7:**
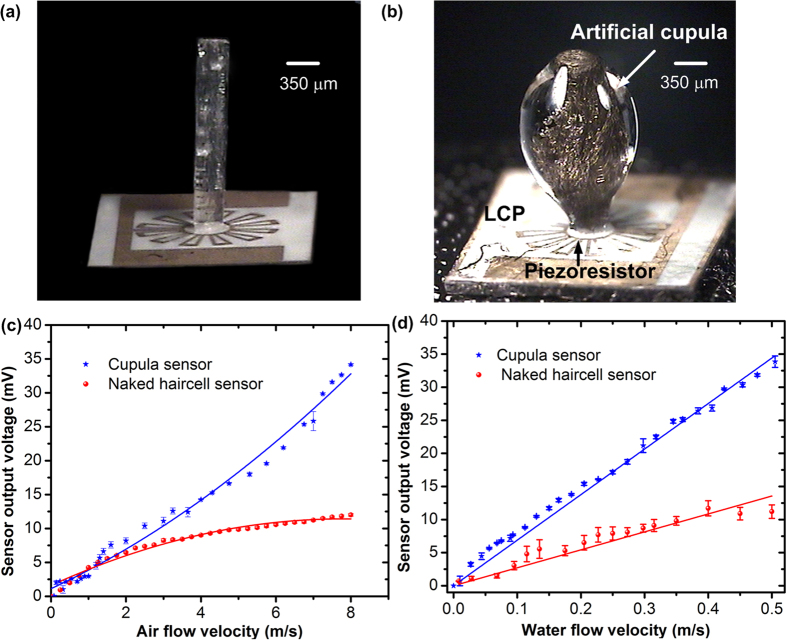
Flow sensing experiments to demonstrate the enhanced sensitivity of the cupula-dressed sensors: (a) Naked hair cell sensor featuring a high-aspect ratio polymer pillar that interacts with the flow. (**b**) Hydrogel-dressed sensor showing the hydrogel cupula and the encapsulated nanofibrils. (**c**) Experimental results of air flow sensing conducted in wind tunnel (**b**) results of the water flow sensing experiments conducted in the water tunnel. The cupula encapsulated sensors showed an enhancement in sensitivity and resolution of flow sensing. (Image (a,b) were taken and edited by Ajay Giri Prakash Kottapalli).

HA-MA solution was drop-casted onto the nanofibril canopy through a 1 ml syringe with a 100 μm diameter needle. The syringe was attached to a precision controlled three-axis micro-positioner that brings the needle right on top of the tip of the hair cell with a gap of a few micrometers. The entire drop-casting process was monitored through a high-speed camera. Spherical droplets of 10 μl were formed at the tip of the needle. As soon as a 10 μl pendant drop was formed at the tip of the needle, it comes into contact with the apex of the pyramid and gets transferred due to the weight of the droplet. 20 drops of HA-MA solution were dropped on the tip of the nanofibril canopy. The HA-MA does not readily seep through the nanofiber matrix due to the hydrophobicity of the PCL nanofibers and creeps slowly over the surface of nanofibrils due to gravity. The drop-casting was repeated on the scaffolds with type i, type ii and type iii fibers (Fig. 6) to determine the fiber diameter that results into an ideal scaffold forming a prolate spheroid shaped hydrogel cupula. Type iii fibers were determined to be too thin and formed a loose network that readily allows the hydrogel to pass through the canopy and reach the LCP membrane. Type i fibers formed a strong and highly dense network that resisted the hydrogel from entering the canopy. This led to accumulation of large quantities of HA-MA solution on the canopy, which caused the network to break due to the weight of the solution. Type ii fibers on the other hand acted as the suitable scaffold in which the fiber network collapsed when the right amount of HA-MA solution was accumulated on it and the entire nanofiber network was later absorbed into the body of the solution. After the drop-casting process, the artificial cupulae were exposed to UV light at 365 nm for 8–10 min and subsequently placed in nanopure water for 12 h to allow complete wetting of the cupula. Swelling to form the hydrogel cupula is an important step because it allows maximum increase in the surface area of the cupula (Fig. 7b and supplementary information S4 show more images of the cupula).

### Experimental flow characterization

An experimental comparative study of the steady-state flow sensing capabilities of the artificial superficial cupula sensor and the naked hair cell sensor, which we developed, was conducted. Separate experiments were conducted by placing both the sensors at the centre of the test-section of the wind and water tunnels to characterize their performance in sensing wind and water flows. The resistance change outputs of the sensors were connected to an external Wheatstone bridge circuit and the resultant voltage output was acquired through data acquisition system and recorded in LABVIEW. The flow velocities in the flow tunnels were varied in steps from the lowest to the highest velocity (0–9 m/s in the wind tunnel and 0–0.5 m/s in the water tunnel).

The flow sensing experiments were repeated several times each on the naked hair cell sensor and the artificial cupula sensor. Experimental results (Fig. 7) show that the artificial cupula sensor demonstrated a sensitivity of 4.34 mV/(m/s) and 77 mV/(m/s) in sensing air and water flows respectively. The small error bars confirm the high repeatability of the sensor output. Addition of the artificial cupula showed an enhancement in sensitivity by 5 times for air flow sensing and 3.5 times for water flow sensing. It was also observed that the sensor demonstrated an increased flow resolution after the addition of the cupula (0.018 m/s as compared to 0.039 m/s for naked hair cell) enabling more data points to be collected during flow sensing experiments with the cupula sensor (Fig. 7d). Flow resolution here is defined as the smallest change in velocity that is distinguishable by the sensor in its voltage output. In the case of water flow sensing, the cupula sensors demonstrated a minimum flow velocity detection threshold of 0.018 m/s.

## Discussion

The nanofibrils ensured a good bonding of the cupula to the hair cells keeping them intact even for water flow velocities beyond 1 m/s. Tiny hair cells that are submerged within the boundary layers generated by the flows do not benefit maximum sensitivity due to sensing within the velocity gradient region. The naked hair cell and the cupula-dressed sensors we developed feature elongated structures that protrude beyond the boundary layers generated by flows for all the experimental velocities. The reason for the enhancement of sensitivity of the cupula-dressed hair cell sensor over the naked hair cell sensor is mainly due to a combination of fluid mechanics of the cupula-fluid interaction and the material properties of hydrogel-like material. The cross-sectional surface area exposed to flow is much higher in case of the cupula encapsulated sensor (which feature a maximum diameter of 1000 μm compared to 350 μm in case of the hair cell), leading to an enhanced drag force. At low Reynold’s numbers, the enhancement of the drag force due to the presence of the prolate shaped cupula can be approximated using the following scaling factor


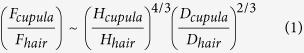


where *F* is the drag force, *H* is the height and *D* is the diameter of the structure. Calculating using the physical geometry of the structures (shown in Fig. 7a,b), the drag force due to the presence of cupula is enhanced by a factor of 2.7 times. Experimental results demonstrated an enhancement in sensitivity by a factor higher than predicated by theory, indicating that there are material factors other than just the change in cross-sectional dimension that contribute to the sensitivity enhancement. The HA-MA network of the cupula is composed of 82% water and plays an important role in the pressure transfer form bulk water to the hair cell. As previously hypothesized^8^,^32^, flexible polymer network of swollen hydrogel material could modify the pressure transfer from flowing water to the encapsulated hair cells. The material’s hydrophilicity and friction component of the water entrapped within the porous hydrogel are believed to be majorly responsible for further enhancement in drag force^32^. Another interesting observation from the experiments was the increase in resolution of the sensor due to the presence of the hydrogel cupula. This indicated that the hydrogel cupula also plays a role in signal filtering. These results are in line with the observations by Peleshenko *et al.*^32^ who also described a reduction in the noise floor in flow sensing experiments with cupula-dressed sensors. The viscous coupling of the cupula and very low relaxation times play a role in suppression of random noise and low frequency noise^42^. Work by biologists in the past also supports the hypothesis revealing that the cupula enhances the signal to noise ratio of the neuromasts by diminishing the effects of Brownian motion of the hair cell^43^. In addition the anticorrosive and antifouling properties of hydrogel enable the sensor to withstand harsh marine environments^44^.

In conclusion, we demonstrated that encapsulation of hair cell sensors inside a biomimetic HA-MA hydrogel cupula enhances the sensitivity and resolution of the flow sensor from 22 mV/(m/s) to 77 mV/(m/s) and 0.039 m/s to 0.018 m/s respectively. We proposed a nanofibril scaffold assisted drop-casting technique that further improves the sensitivity of these cupula sensors. The nanofibril scaffold enhances the drag force by ensuring the exposure of cupula’s maximum surface area to the flow in regions beyond the velocity gradient that occurs within the boundary layer of the flow. The high sensitivity of the sensor is observed to be due to a combination of factors – enhanced drag force due to the increased cupula surface area, hydrogel material enforced drag force enhancement, LCP sensing membrane, and protrusion of the cupula beyond the flow-generated boundary layers. Low-powered, low-cost and miniaturized sensors reported in this work can be used in the navigation and maneuvering of underwater robots, but can also find applications in biomedical and microfluidic devices. The developed sensors meet the stringent demands on high sensitivity and low threshold detection limits that are required for intravenous flow monitoring. Arrays of these artificial cupula sensors could replicate the functionality of a lateral-line of fish on URVs and thus aid in useful strategies like energy-efficient maneuvering, artificial vision, improved control and maneuverability of the vehicle. The sensors can detect flow separation on the hulls of the underwater vehicles, and sense minute flow velocities within the vortices, which can bring a sea change in improving the vehicle maneuverability. Furthermore, the fabrication approach demonstrated here can potentially guide towards the development of other biomimetic sensors, structures and materials in the future. Such biomimetic sensors would also facilitate fundamental study of biomimetic sensors, assist biologists to understand the locomotory and detection mechanisms in fishes.”

## Methods

### Modification of HA

HA was modified following methods described previously^45^. HA powder was dissolved in de-ionized (DI) water and stirred overnight at room temperature to prepare 1.5% (w/v) solution. The following day, pH of the HA solution was adjusted to 8 using 5 M NaOH while slowly adding 20 molar excess of MA (methacrylic anhydride) to it. After addition of MA, HA was allowed to react with it for 2 h by maintaining the pH at 8. The HA-MA solution was stored at 4 °C for 24 h. It was then dialyzed against large volume of 0.1 M NaCl solution using a dialysis bag (10 kD MWCO, 2.9 cm diameter, flat width 4.5 cm) for 48 h and then against alternating solutions of 1:4 EtOH–H O (v/v) and pure H_2_O for 24 h. Finally, the HA-MA solution was lyophilized for 72 h.

### Electrospinning to form the nanofibril scaffold

Nanofibers were formed from a solution of polycaprolactone (PCL) (80,000 MW) in chloroform (CHCl_3_), 11.7% by weight. Three different types of scaffolds were formed from the same precursor solution but at three different electrospinning process conditions. The hair cell sensor was placed on an aluminium substrate and positioned under the syringe needle at a distance of *h* from the tip of the needle. The electrospinning process was optimized to ensure that the fibers first encounter the standing pillar and then touch the sensor base. This avoids the fibers from being deposited directly on the sensor membrane. When the voltage applied reaches a critical value, the electrostatic force overcomes the surface tension of the Taylor cone that was suspended at the tip of the needle, and a jet starts to emerge from the tip of the cone. The electrostatic forces cause extensive stretching of the jet before it is collected on the substrate and therefore, the diameters of the fibers significantly reduce. During the stretching process, the solvent in the fibers gets evaporated and this leads to further reduction of the fiber diameters.

### Drop casting

HA-MA solution was collected into a syringe which was attached to a XY position controllable stage. The Z-axis was manually adjusted in such a way that the needle tip of the syringe (of diameter 100 μm) was brought just above the hair cell. Droplets of approximately 10 μl were dispensed each time over the hair cell. The entire drop-casting process was observed through a high-speed camera that was set to view at a 90^o^ angle. The needle of the syringe was carefully aligned on top of the hair cell in such a way that the hair cell stands pointing towards the center of the spherical droplet that was formed at the tip of the needle. In case of any misalignment, the cupula formed will be asymmetric around the hair cell and the asymmetricity amplifies when the cupula swells during the wetting process. Occasionally, precipitate would form at the tip of the syringe due to evaporation of the electrolyte leading to a viscous droplet sticking at the exit of the needle. The needle was replaced from point-to-point during the process. This process is standardized and is found to be quite repeatable from sensor to sensor. The entire process involved dropping about 20 droplets of hydrogel.

### Wind tunnel and Water tunnel experiments

A custom-made open circuit wind tunnel with a test section of dimensions 0.4 m(W) × 0.4 m(H) × 2 m(L) was used for the experiment. A commercial hot-wire anemometer flow sensor was used to calibrate the flow velocity in the wind tunnel. Water flow experiments were conducted in Long Win LW-3457 model circulating water tunnel. The water tunnel has a test section of dimensions 0.3 m(W) × 0.4 m(H) × 1 m(L). Turbulence reducing steel screens and honeycomb layers ensure a steady-state flow within the tunnel for a large range of velocities from 0–0.6 m/s. A Pitot tube set-up was used to calibrate the flow velocity inside the water tunnel. Flow inside the wind and water tunnels was regulated by controlling the frequency rpm of the motor that drives the fluid.

## Additional Information

**How to cite this article**: Kottapalli, A. G. P. *et al.* Nanofibril scaffold assisted MEMS artificial hydrogel neuromasts for enhanced sensitivity flow sensing. *Sci. Rep.* **6**, 19336; doi: 10.1038/srep19336 (2016).

## Supplementary Material

Supplementary Information

## Figures and Tables

**Figure 1:**
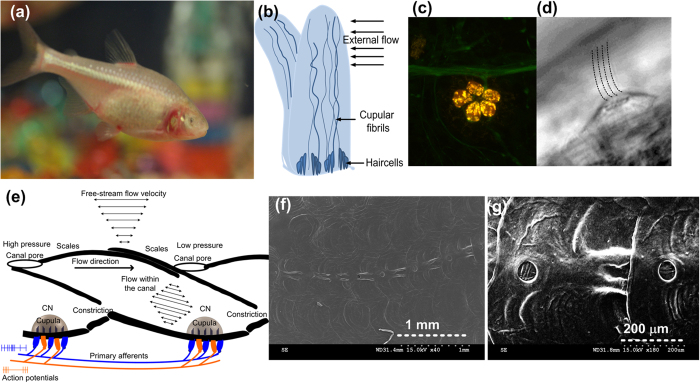
Bioinspiration - neuromast sensors in the blind cavefish: (**a**) Photograph of the blind cavefish that live in lightless environments, hence relying on distributed arrays of neuromast sensors for hydrodynamic flow imaging and navigation. (**b**) A schematic of the cupula showing the basic morphology of the biological neuromast sensor. (**c**) Confocal laser scanning microscopy image, showing the hair cells of a neuromast stained in red and the lateral line nerve stained in green. (**d**) Microscopic DIC image of a superficial neuromast of the larvae zebra fish seen in lateral view. (**e**) Schematic showing the basic morphology of the canal neuromasts. (**f**) A scanning electron microscope (SEM) image of canal pores that exist on the opercula region of the blind cave fish. A single neuromast exists embedded within the canal at the center of the canal region between the pores. (**g**) Zoomed-in view showing the canal pores closely. (Images (**a**,**b**,**e**,**f**,**g**) were taken by Mohsen Asadnia and Ajay Giri Prakash Kottapalli. Images (**c,d**) were taken by Melanie Haehnel from the *The Whitney’s laboratory for Marine Bioscience, St. Augustine, Florida*. Permissions have been granted for the usage of images (**c,d**). Images were edited by Ajay Giri Prakash Kottapalli).

**Table 1:** Electrospinning parameters to form three types of fibers with three different diameters.

Type	Needle diameter (Gauge)	Applied voltage (kV)	Feed rate (ml/h)	Spinning time (min)
Type i	18	10	1.2	15
Type ii	25	15	0.5	20
Type iii	25	22	0.5	5
