# Supplementary material for: Nanofibril scaffold assisted MEMS artificial hydrogel neuromasts for enhanced sensitivity flow sensing
Source: Sci Rep. 2016 Jan 14;6:19336. doi: 10.1038/srep19336 (PMC4725914; doi:10.1038/srep19336)
Supplement: Supplementary Information [file srep19336-s1.docx]

**Nanofibril scaffold assisted MEMS artificial hydrogel neuromasts for enhanced sensitivity flow sensing**

Ajay Giri Prakash Kottapalli^1,2*^, Meghali Bora^3^, Mohsen Asadnia^1,2^_,_ Jianmin Miao^1^, Subbu S Venkatraman^3^, Michael Triantafyllou^2,4^

^1^School of Mechanical & Aerospace Engineering, Nanyang Technological University,

50 Nanyang Avenue­­­­, Singapore 639798

^2^Center for Environmental Sensing and Modeling (CENSAM) IRG

Singapore-MIT Alliance for Research and Technology (SMART) Centre, 3 Science Drive 2, Singapore 117543

^3^School of Material Science and Engineering, Nanyang Technological University,

50 Nanyang Avenue­­­­, Singapore 639798

^4^Department of Mechanical Engineering, Massachusetts Institute of Technology,

77 Massachusetts Avenue, Cambridge, MA 02139

^*^Corresponding author (Email): ajay_k@mit.edu, (Tel): +65 65165702, (Fax): +65 67906995

**Supplementary information S1: Hair cell design and boundary layer analysis**

The minimum height of the hair cell is limited by the boundary layer generated during flow sensing. The flow velocity within the boundary layer is lower than the overall bulk flow velocity and tiny hair cells fabricated in the past^32^, that are submerged within the boundary layer do not measure the actual bulk flow velocity. Moreover, taller hair cells with high aspect ratios increase the sensitivity of the sensor. Although a comprehensive treatment of the fluid dynamics aspect is beyond the scope of this paper, a simple relationship can be used to determine the height of the boundary layer that the hair cell faces. If we denote the main stream flow velocity as *U*, distance from the leading edge of the plate *x*, Reynolds number based on the free-stream velocity and the length along with plate *Re_x_* (*Re_x_* = *Ux/ʋ*), where *ʋ* is the kinematic viscosity of the fluid, then *δ* the outer limit of the boundary layer (boundary layer thickness) which occurs at

for laminar flow:

 (1)

and for turbulent flow:

(2)

In the case of our sensor, the distance from the leading edge (*x*) was 2 mm. For airflow velocity of 10 m/s (highest flow velocity for which the sensor was tested), and 0.05 m/s (lowest flow velocity for which the sensor was tested), the Reynolds numbers (*Re_x_*) were 1276 and 6, respectively and the boundary layer heights were 280 μm and 2.8 mm respectively. For water flow velocity of 0.5 m/s (highest flow velocity for which the sensor was tested), and 0.018 m/s (lowest flow velocity for which the sensor was tested), the Reynolds numbers are 1250 and 45 respectively and the heights of the boundary layer are 280 μm and 1.5 mm respectively. Therefore, the boundary layer thickness poses a limitation on the minimum height of the hair cell for the flow sensor. In order to address this issue, unlike tiny SU-8 hair cells developed in the past, in this work, we developed fabricated taller hair cells through stereolithography process.

In order to further confirm the design of the heir cell dimensions, we conducted finite element simulations to optimize the height and the diameter of the hair cell that maximize the sensitivity of the sensor while being feasible to be fabricated. Simulations were conducted with the hair cell sensor placed in a constant dc flow velocity of 0.5 m/s for various hair cell heights and diameters. Initially, the diameter of the hair cell was fixed at a constant value and the displacement profile of the LCP membrane was simulated for various hair cell heights. The maximum displacement of the membrane initially increased linearly with the height of the hair cell but started to saturate at even taller hair cell dimensions above 2700 μm. Moreover, the difference in maximum displacement of the membrane started to increase at 2700 μm and above. Therefore, considering the boundary layer analysis and the FEA simulation analysis, the height of the hair cell was chosen to be 2700 μm.

**Supplementary information S2: Chemical structure of HA-MA Hydrogel:**


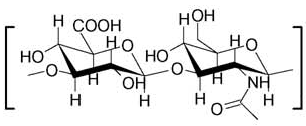


HA


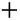


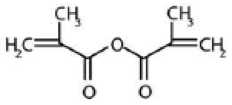


MA


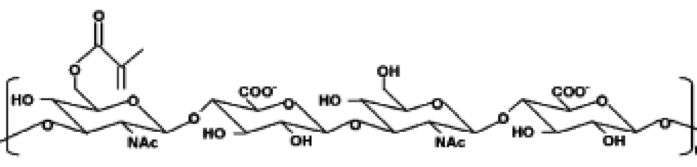


HA-MA

The chemical structure is modified from^45^ **(Copyrights have been procured from the publisher)**

**Supplementary information S3: Calculation of swelling ratio and other parameters:**

Swelling study was conducted in water at room temperature to estimate the network structure parameters of hydrogels using the Flory-Rehner calculations as described previously^41^. Firstly, volumetric swelling ratio, *Qv,* was calculated from the following equation:

$$Q_{v}=1+ \frac{\rho_{p}}{\rho_{s}} \left( Q_{m}- 1 \right) (1)$$

where ρ*_p_* is density of polymer (1.229 g/cm^3^) and ρ*_s_* is density of solvent (1 g/cm^3^ for water). *Q_M_* is mass swelling ratio and was obtained from the swelling ratio experiment.

Next, *Qv* was used to estimate the average molecular weight between crosslinks,$\bar{Mc},$ using the following simplified version of Flory-Rehner equation:

$${Q_{v}}^{5/3}= \frac{\bar{v} \bar{Mc}}{V_{1}} \left( \frac{1}{2}- \chi\right) (2)$$

where $\bar{v}$is specific volume of polymer, *V*_1_ is molar volume of solvent (18 mol/cm^3^ for water), and χ is polymer-solvent interaction parameter (0.473 for HA).

After $\bar{Mc}$was estimated, the effective crosslink density, $v_{e}$, was determined from the following equation:

$$v_{e}= \frac{\rho_{p}}{\bar{M_{c}}} (3)$$

The water swollen hydrogels mesh size, ξ, was calculated by the following equation:

$$\xi= {Q_{v}}^{1/3} \sqrt{{\bar{r_{o}}}^{2}} (4)$$

where $\sqrt{{\bar{r_{o}}}^{2}}$is the root-mean square distance between crosslinks, previously reported as the following equation for HA:

$$\sqrt{\left( \frac{{\bar{r_{o}}}^{2}}{2n} \right)} \cong2.4 nm (5)$$

where *n* is the number of monomer units for HA. For HA with a molecular weight (*M_n_*) of ~ 1 × 10^6^, *n* is ~2494 which gives the following estimation of$\sqrt{{\bar{r_{o}}}^{2}}$:

$$\sqrt{\bar{{r_{o}}^{2}}}=0.1695 \sqrt{M_{n}} (nm) (6)$$

Finally, the mesh size can be calculated by combining equations (4) and (6) as follows:

$$\xi= 0.1695 \sqrt{\bar{M_{c}}} {Q_{v}}^{1/3} (nm) (7)$$

Estimated values of all swelling study parameters, *Qm*, *Qv,*$\bar{Mc},$*v_e_*, and ξ should be considered as approximate values on account of certain assumptions considered in Flory-Rehner calculations. These values, however, can be used for making comparisons between various network features of different HA hydrogels.

**Supplementary information S4: Additional schematics, microscope and SEM images**


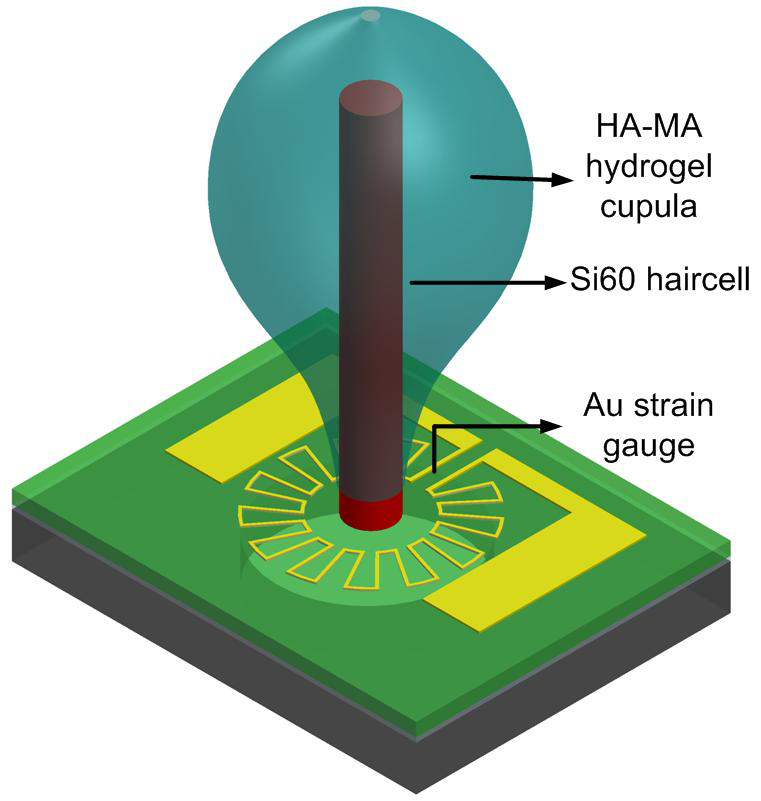


Schematic of the sensor structure (not drawn to scale)


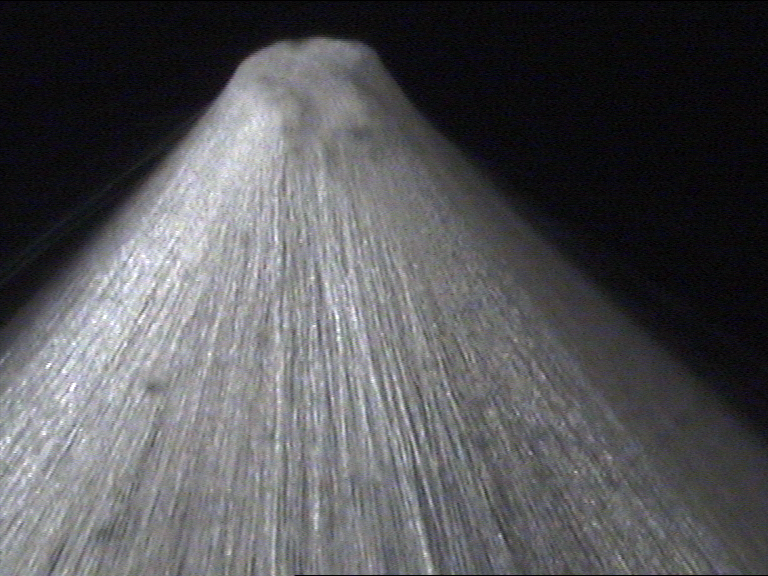


Aligned PCL nanofibers formed by electrospinning on the hair cell sensor. **This optical image is taken by Ajay Giri Prakash Kottapalli**


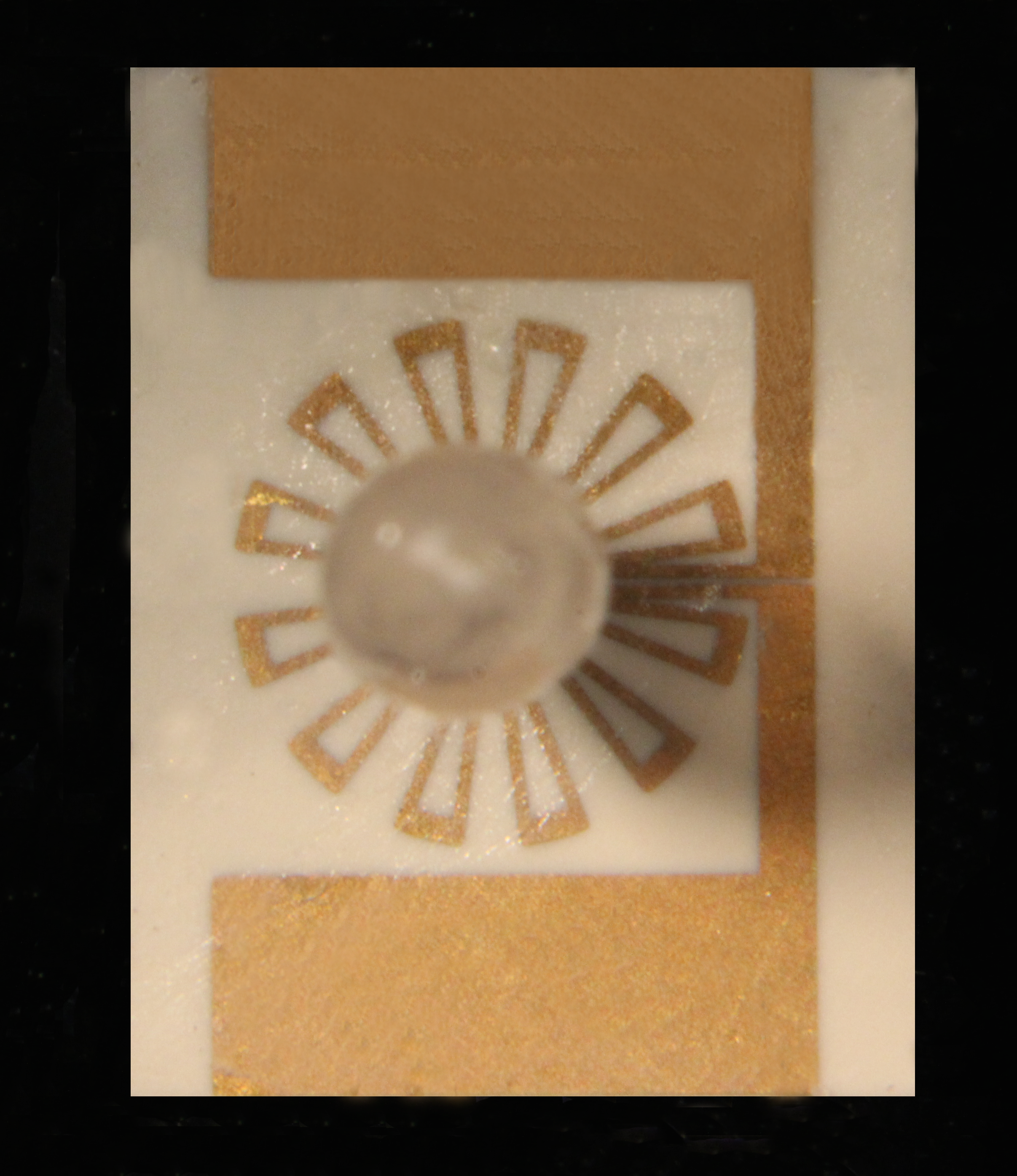


Optical microscope image of the top-view of a prolate spheroid shaped HA-MA cupula located at the center of the LCP membrane. **This optical image is taken by Ajay Giri Prakash Kottapalli**

**References**

[32] Peleshenko, S *et al.* Hydrogel-encapsulated microfabricated hair cells mimicking fish cupula neuromast. *Adv. Mater.* **19**, 2903-2909 (2007).

[41] Leach, J. B., Bivens, K. A., Patrick, C. W. & Schmidt, C. E. Photocrosslinked hyaluronic acid hydrogels: Natural, biodegradable tissue engineering scaffolds. *Biotechnol. Bioeng.* **82**, 578-589 (2003)

[45] Smeds, K. A. *et al.* Photocrosslinkable polysaccharides for in situ hydrogel formation. *J. Biomed. Mater. Res.* **54,** 115-121 (2000).
